# Transcriptome analysis of genes associated with breast cancer cell motility in response to Artemisinin treatment

**DOI:** 10.1186/s12885-017-3863-7

**Published:** 2017-12-15

**Authors:** Kanchan Kumari, Sunita Keshari, Debomita Sengupta, Surendra C. Sabat, Sandip K. Mishra

**Affiliations:** 1 Cancer Biology Laboratory, Institute of Life Sciences (Government of India), Nalco Square, Bhubaneswar, Odisha 751023 India; 2 National Central University, Chungli, Taiwan; 3 Molecular biology of abiotic stress, Institute of Life Sciences, Nalco Square, Bhubaneswar, Odisha 751023 India; 4 Department of Molecular Medicine, Bose Institute, Kolkata, India

**Keywords:** Breast cancer, Proliferation, Migration, Invasion, HDACs

## Abstract

**Background:**

Well-known anti-malarial drug artemisinin exhibits potent anti-cancerous activities. In-vivo and in-vitro studies showed its anti-tumor and immunomodulatory properties signifying it as a potent drug candidate for study. The studies of mechanisms of cell movement are relevant which can be understood by knowing the involvement of genes in an effect of a drug. Although cytotoxicity and anti-proliferative activity of artemisinin is evident, the genes participating in its anti-migratory and reduced invasive effect are not well studied. The present study reports the alteration in the expression of 84 genes involved in cell motility upon artemisinin treatment in MCF-7 breast cancer cells using pathway focused gene expression PCR array. In addition, the effect of artemisinin on epigenetic modifier HDACs is studied.

**Methods:**

We checked the functional stimulus of artemisinin on cell viability, migration, invasion and apoptosis in breast cancerous cell lines. Using qRT-PCR and western blot, we validated the altered expression of relevant genes associated with proliferation, migration, invasion, apoptosis and mammary gland development.

**Results:**

Artemisinin inhibited cell proliferation of estrogen receptor negative breast cancer cells with fewer efficacies in comparison to estrogen receptor positive ones. At the same time, cell viability and proliferation of normal breast epithelial MCF10A cells was un-affected. Artemisinin strongly inhibited cancer cell migration and invasion. Along with orphan nuclear receptors (ERRα, ERRβ and ERRγ), artemisinin altered the ERα/ERβ/PR/Her expression status of MCF-7 cells. The expression of genes involved in the signaling pathways associated with proliferation, migration, invasion and apoptosis was significantly altered which cooperatively resulted into reduced growth promoting activities of breast cancer cells. Interestingly, artemisinin exhibited inhibitory effect on histone deacetylases (HDACs).

**Conclusions:**

Upregulated expression of tumor suppressor genes along with reduced expression of oncogenes significantly associated with growth stimulating signaling pathways in response to artemisinin treatment suggests its efficacy as an effective drug in breast cancer treatment.

## Background

Breast cancer, despite of early detection, new discoveries and increased awareness, remains the second leading cause of cancer related deaths in women worldwide. Although genetic and hormone oestrogen are the most important risk factors for breast cancer, factors like high iron content significantly contribute towards tumorigenesis [1–6]. Studies suggest strong co-relation of iron with various cancers [7–10]. Increased and decreased iron content in post and premenopausal women has been explored to be associated with increased breast cancer risk through pathways like oxidative stress and angiogenesis respectively. Wormwood (*Artemisia annual*) plant derived extract artemisinin is chemically a sesquiterpene lactone with a 1,2,4-trioxane ring system. The endoperoxide moiety of artemisinin forms free radicals on reaction with iron that is essential for cell division and proliferation. Compared with non-cancerous cells, depending on the tumor aggressiveness, cancer cells have a higher number of cell surface transferrin receptors, which pick up iron via interaction with the plasma iron-carrying protein transferrin. By virtue of a higher rate of iron uptake, cancer cells would be selectively more vulnerable to the cytotoxicity of artemisinin [11, 12]. Natural products such as artemisinin and many other have been tested for their cytotoxic effect on breast cancer cells [13–15]. Various in-vitro and in-vivo studies have been done to investigate the role of transferrin and its conjugates in iron-mediated effect of artemisinin in breast cancer [16–20]. Artemisinin derivatives and compounds bearing skeleton of artemisinin have also been investigated for their anti-cancerous effects [21–27]. Various nano-formulations of artemisinin is tested for effective artemisinin targeting breast cancer both in-vitro and in-vivo [28–36]. Also, combinational therapies have been done to study and compare the synergistic effect of artemisinin in breast cancer [37–42]. In-vivo studies show the potential benefits of artemisinin in breast cancer treatment [43–50] Pharmacokinetics and toxicity of artemisinin has also been tested in breast cancer patients during phase-I study [51–53]. Mechanisms underlying artemisinin-mediated anti-proliferative and apoptosis inducing role in breast cancer have also been explored [54–63]. Role of artemisinin in drug resistance has been studied as well [64, 65].

Role of transcription factor E2F and its target genes in the anti-proliferative activity of artemisinin in breast cancer is reported [57]. In present study, we first checked the effect of artemisinin treatment on cancer cell viability, proliferation, migration, invasion and apoptosis. We then report the involvement of relevant genes in the respective signaling pathways in an effect of artemisinin treatment. Taken together, our results demonstrate the molecular basis of anti-proliferative, migratory, invasion and apoptosis inducing effect of artemisinin in breast cancer. Also for the first time we have reported the HDAC inhibitory effect of artemisinin.

## Methods

### Drug

Artemisinin (C15H22O5) was a kind gift from IPCA (International pharmaceutical company, Mumbai, India). The stock solution of artemisinin was prepared in Dimethyl Sulfoxide (DMSO). The final DMSO concentration during treatment in the culture medium was maintained below 0.01%.

### Cell culture

Breast cancer cell lines MCF-7, T47D and MDA-MB-231 were purchased from National Centre for Cell Sciences (NCCS), Pune, India. The MCF-7 cells were cultured in Dulbecco’s Modified Eagle’s Medium (DMEM) whereas T47D and MDA-MB-231 cells in Roswell Park Memorial Institute medium (RPMI) supplemented with 10% fetal bovine serum (FBS) and penicillin-streptomycin (MP Biomedicals) at 37 °C, 5% CO_2_ and 95% humidity. MCF10A, a kind gift from Dr. Annapoorni Rangarajan (IISC, Bangalore, India) was maintained in DMEM F12 containing horse serum supplemented with hydrocortisone, EGF, insulin, cholera toxin and penicillin-streptomycin at 37 °C, 5% CO2 and 95% humidity. The cells were grown until 70-80% confluence and then sub cultured with Trypsin-EDTA. All experiments involving treatment were performed in cells kept in phenol red free medium containing charcoal treated fetal bovine serum supplemented with penicillin-streptomycin for 48 h.

### Cell viability assay

The effect of artemisinin on viability of cells was checked by 3-(4,5-Dimethylthiazol-2-yl)-2,5 Diphenyltetrazolium Bromide (MTT) assay. MCF10A, MCF-7, T47D and MDA-MB-231 cells were seeded at a density of 3 × 10^3^ cells/well in 96 well plates. The cells were treated with different concentrations of artemisinin (500 nM, 1, 10, 50 and 100 μM) and incubated for two different time periods (12 and 24 h). After appropriate time period, 10 μL of MTT (MP Biomedical) (5 mg/mL in PBS) was added into each well and incubated at 37 °C, 5% CO_2_ atmospheric condition for another four hours. After incubation, the medium was removed and 100 μL of DMSO was added to dissolve thus formed formazan crystals. The solubilized crystals were then quantified by scanning the plates at 570 nm using Varioskan™ Flash Multimode Reader (Thermo Scientific). Three independent sets of experiments were performed to evaluate the effect of artemisinin. The percent viability was calculated by the formula-.

% viability = A/A_0_ X 100 where A_0_ and A are the absorbance of vehicle control and artemisinin treated cells respectively.

The IC_50_ value of artemisinin was calculated for different cell types using the nonlinear regression curve fit XY analysis of GraphPad prism software.

### Colony forming assay

For colony forming assay 0.6 X 10^3^ of MCF10A, MCF-7, T47D and MDA-MB-231 cells were seeded in triplicates in 12-well plate (Falcon Becton Dickinson) and after 24 h of cell attachment, the cells were treated with 1 μM of artemisinin. The plates were under incubation for 10 days at 37 °C, 5% CO_2_ to allow the growth of colonies (~50 cells/ colony). During long-term incubation, fresh complete growth medium with 1 μM of artemisinin was replaced after every three days. The cells were washed twice with 1X PBS (137 mM NaCl, 2.7 mM KCl, 10 mM Na_2_HPO_4_ and 2 mM KH_2_PO_4_), fixed with 10% (*v*/v) formalin and then stained with 0.01% (*w*/*v*) crystal violet solution. The excess stain was removed by washing with 1 X PBS. The plate was air-dried and image was captured using Gel Doc™ XR + Imager (Bio-Rad). To quantify the rate of colony formation, the stained cells in the form of colonies were dissolved in 10% (v/v) acetic acid and the absorbance was quantified at 540 nm using Varioskan™ Flash Multimode Reader (Thermo Scientific). The values is presented using the formula-.

Colony formation rate = 100% X (experimental absorbance value / control absorbance value).

### Wound healing assay

1 X 10^4^ MCF-7 cells were plated and grown up to 90% confluence in 12-well plate (Falcon Becton Dickinson). To restrict proliferation and to study only the migration of cancerous cells, the plated cells were kept in serum free media for 48 h. Cells were then scratched with a sterile 200 μL pipette tip (two vertical and two horizontal lines) in each well. The cells were washed twice with 1X PBS and the image was captured such as cells at stage 1 that is 0 h. Cells were treated with 1 μM artemisinin. Images of the cells undergoing migration were then taken at different time points at a magnification of 4X. Quantitation of migrated cells was done by calculating the decrease in area at all the observed time points with the help of ImageJ software.

### Transwell migration and invasion assay

Transwell-migration assay was performed following manufacturer’s protocol (BD Falcon, USA). Appropriately artemisinin (1 μM, 72 h) treated MCF-7 cells were seeded at a density of 2.5 × 10^4^ cells in upper chamber of 12 well transwell system in 500 μL of serum and phenol red free DMEM. Medium supplemented with 5% serum was used as chemoattractant in the lower chamber. After 24 h the cells on both side of the membrane were fixed with 10% formalin and stained with 0.01% crystal violet stain. The cells were scrubbed on the seeded side to quantify the percent of migrated cells only. The membrane was then washed with PBS and the cells attracted towards the serum were visualized under light microscope and pictured (10X) under different field views. The number of migrated cells in control and artemisinin treatment in 10 different fields was calculated using ImageJ software and the average value was represented in the graph. For invasion assay, the transwell migration chamber was coated with matrigel (2 mg/ml) (BD Biosciences). The cells present towards the lower side of the chamber were considered as invaded cells and were fixed and stained with crystal violet dye similar to migration assay.

### Apoptosis detection assay

MCF-7 cells were seeded at a density of 5 X 10^4^ cells/well in 35 mm plates. Further the cells were treated with 1 μM artemisinin and incubated for 24 h at 37 °C in 5% CO_2_. Cells were stained using a PE Annexin V Apoptosis Detection Kit (BD Pharmingen, San Diego, CA, USA) according to the manufacturer’s protocol. Acquisition was performed using BD FACS Calibur (San Jose, CA, USA). 1X10^4^ cells were analyzed using FL3 filter for 7-AAD-positive cells and FL2 filter for the PE-annexin V-positive cells. Plumbagin (5-hydroxy- 2-methyl-1, 4-naphthaquinone) was taken as positive control due to its role in induction of apoptosis at higher rate [66].

#### qRT-PCR array

The Human Cell Motility RT2 Profiler PCR Array purchased from Qiagen was employed to study the effect of artemisinin on genes associated with movement of cells. The array contained 84 genes including genes associated with development, growth factors, receptors important for chemotaxis and mobilization. Total RNA was isolated using Trizol from appropriately artemisinin (10 μM,72 h) treated and control MCF-7 cells. Equal amount of properly DNase I treated RNA was used to prepare cDNA using first stand cDNA synthesis kit (Invitrogen). Real time assay was performed with the array plate. mRNA level and fold change for each gene compared to control was calculated using value of cycle threshold. The alteration in the expression of genes was validated by qRT PCR and/or western blot assay. β2 microglobulin and 18S was used for normalization.

### Western blot analysis

For western blot whole cell lysate of appropriately treated cells was prepared using RIPA buffer [20 mM Tris-HCl (pH 7.5), 150 mM NaCl, 1 mM Na_2_EDTA, 1 mM EGTA, 1% NP-40, 1% sodium deoxycholate, 2.5 mM sodium pyrophosphate, 1 mM β-glycerophosphate, 1 mM Na_3_VO_4_ and 1 μg/mL leupeptin]. The lysed samples were collected after centrifugation for 30 min at 12,000 rpm, 4 °C. Equal amount (40 μg) of protein was loaded after Bradford method of protein quantification. The samples were run in 10% SDS-PAGE gel, transferred on PVDF membrane (Millipore) and blocked with 5% (*w*/*v*) non-fat milk (Sigma). Blots were then incubated with primary antibody overnight [Cytochrome c (1:5000), p21 (1:5000), β catenin (1:5000), α-tubulin (1:1000), Bcl2 (1:1000), caspase 9 (1:1000, Cell Signaling), p53 (1:500, Calbiochem), p21 (Cell signaling 1:1000), E-cadherin(Cell signaling 1:1000) and HDACs (1:1000, HDAC Ab Sampler Kit, Cell Signaling)]. Thereafter, 1 h with their respective HRP conjugated secondary antibody [anti rabbit (1:5000, Sigma Aldrich) or anti mouse (1:5000, Sigma Aldrich)], the blots were then subjected to chemilumenescent detection reagent (GE Healthcare) for visualization and the bands were detected by using Gel Doc™ XR + Imager*.* Densitometric analyses of the protein bands was calculated by using ImageJ software.

### Immunofluorescence

Cells at a density of 3 X 10^4^ were grown in 0.2% gelatin coated coverslips in 35 mm plates. The 10 μM artemisinin treated cells were washed with ice-cold 1X PBS, fixed with methanol:acetone (1:1) and kept at -20 °C for 30 min-1 h. The cells were then blocked with blocking buffer [0.1% (w/v) bovine serum albumin, 0.3% (*v*/v) Triton™ X-100 in 1X PBS] for 2 h and then incubated with primary antibodies [Cytochrome c antibody (1:500, Santa Cruz), β catenin (1:5000)] overnight at 4 °C. Next day the cells were washed with TBST (1X TBST: 50 mM Tris.HCl, pH 7.4, 150 mM NaCl, 0.1% Tween 20.), then incubated with flourocrome conjugated anti-mouse antibody (1:1000, Alexa Fluor® 594, Life Technologies) for 1 h. The cells were then washed with TBST and further incubated with DiOC6 (3,3′-Dihexyloxacarbocyanine Iodide), a mitochondrial stain (1:1000, Life Technologies). Finally the coverslip was mounted on a slide using Prolong ® Gold Antifade Reagent (Life Technologies) and the images were captured using confocal microscope (Leica Microsystems CMS GmBH, Mannheim, Germany) using LAS AF application suite (Leica Application Suite Advanced Fluorescence).

### Statistical analysis

Data analysis was performed by unpaired t test or by one way ANOVA using GraphPad Prism^*®*^ software where the *p*-values *≤*0.05 were considered as significant.

## Results

### Reduced cell growth and colony formation of breast cancer cells upon artemisinin treatment

**Fig. 1:**
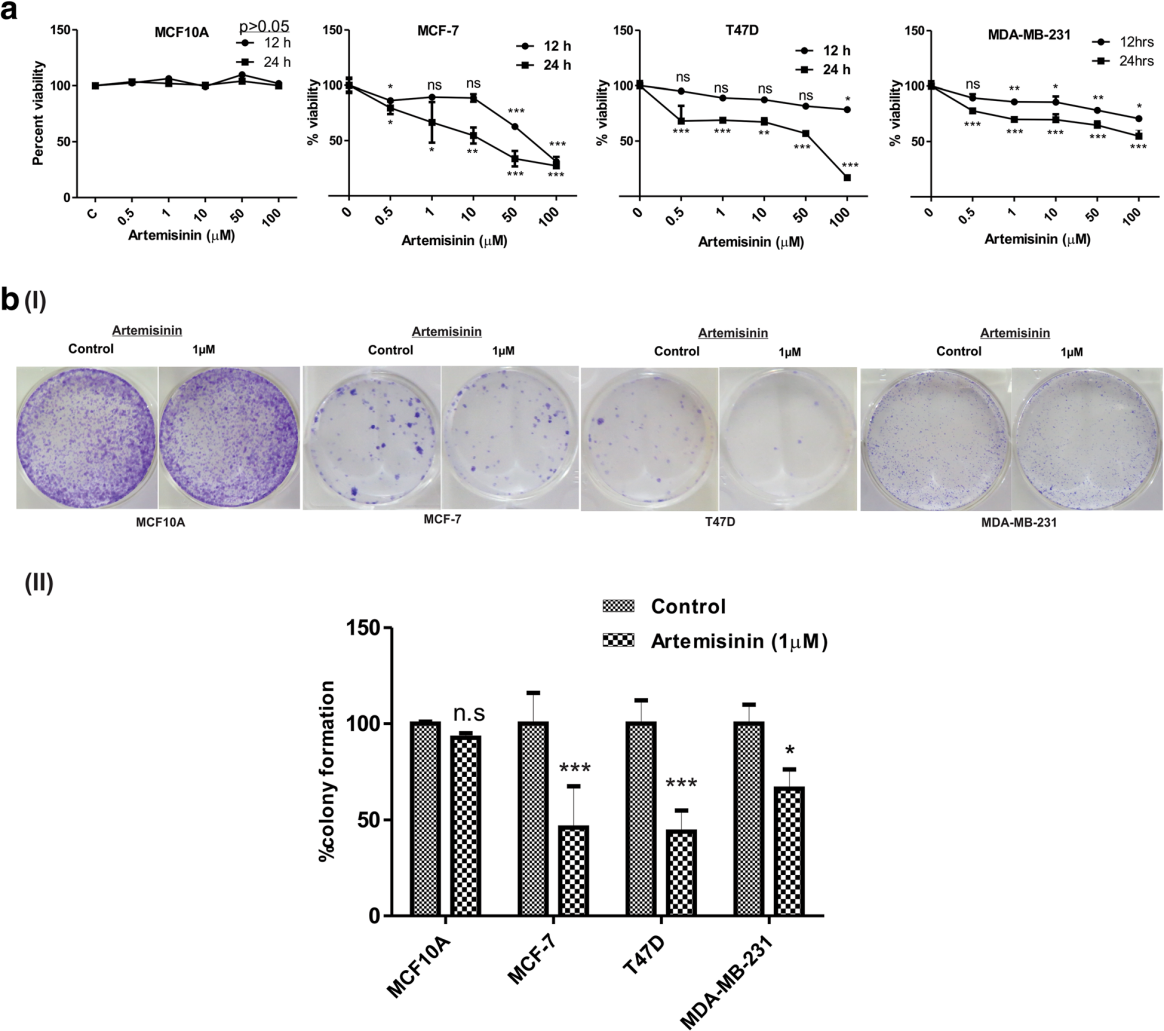
Artemisinin inhibits growth and colony forming ability of estrogen receptor positive breast cancer cells. (A) Viability assay in MCF10A, MCF-7, T47D and MDA-MB-231 breast cancer cells showing the effect of artemisinin treatment in a dose and time dependent manner where artemisinin concentration is indicated in X axis and percentage viability compared to control is indicated on the Y axis. The mean + SEM for three independent experiments was calculated. Statistically significant difference was found between the absorbance of control and artemisinin treated samples ***p (<0.001), **p (<0.0078) and ns *p* (>0.05). B (I) Representative image of colony forming assay of artemisinin treated MCF10A, MCF-7, T47D and MDA-MB-231 breast cancer cells. (II) Graph represents mean + SEM of control, and treated samples in three separate experiments performed in triplicate, *p(<0.05), ***p (<0.001)

Cell viability assay performed using MTT suggested that during the initial stage of artemisinin treatment (12 h) there is no significant reduction in viability of the cells but after 24 h of artemisinin treatment the viability of both MCF-7 and T47D breast cancer cells was inhibited in a dose dependent manner (Fig. 1A). The effect of artemisinin as found in the breast cancer cells was not observed in normal breast epithelial cells MCF10A. When the assay was carried out in triple negative MDA-MB-231 breast cancer cells, the reduction in viability of the cells was reduced and was found to be less effective in the same range. IC_50_ value of artemisinin upon 24 h of treatment was found to be 60.55 μM, 32.14 μM and 88.08 μM for MCF7, T47D and MDA-MB-231 respectively. 1 μM dose of artemisinin which is physiologically relevant was used in further experiments. Next to explore the effect of artemisinin on proliferation of breast cancer cells as well as normal breast epithelial cells, clonogenic assay was performed. On the day of harvest, 50% inhibition in colony formation was observed in 1 μM artemisinin treated both MCF-7 and T47D cells while MCF10A cells remained unaffected. In MDA-MB-231 cells, 36% less colonies were detected upon artemisinin treatment (Fig. 1B).

### Artemisinin restricted breast cancer cells migration & invasion and induced apoptosis

**Fig. 2:**
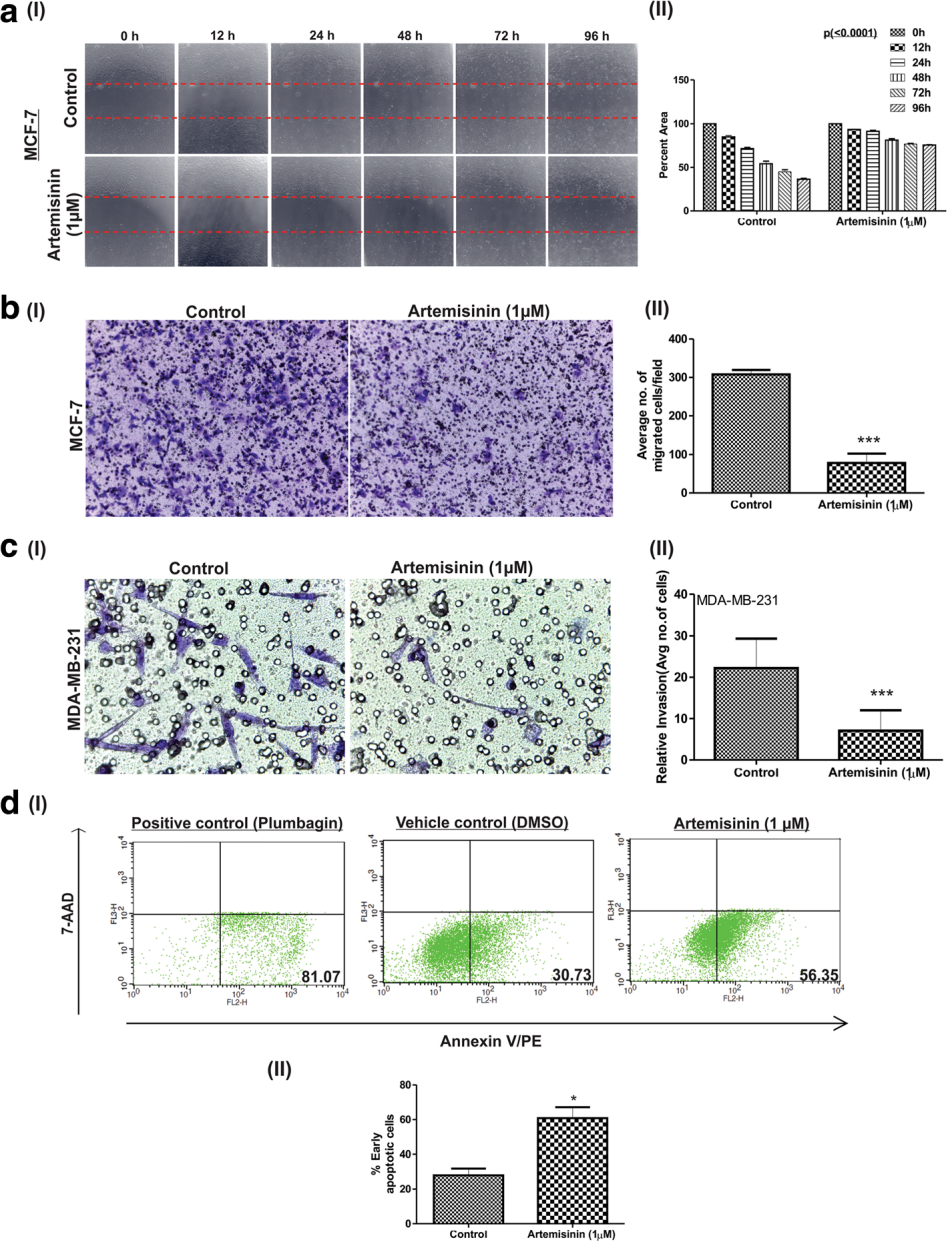
Artemisinin exhibits anti-migratory, anti-invasion and apoptosis inducing property in breast cancer cells. A (I) Picture represent relative cell migration in both control and treated MCF-7 cells at different time intervals. (II) Graph represents the quantification of the decrease in the area as wound healing progresses at the observed time points. Significant differences were observed between control and treated cells at different time points *p* (<0.0001). B (I) Image depicts the cell migration in control and artemisinin treated MCF7 cells as observed in transwell migration assay. (II) Graph depicts the average number of migrated cells. C (I) Diagram represents relative invasion in control and artemisinin treated aggressive breast cancer cells. (II) Relative invasion in depicted in the graph. D (I) Dot plot representing PE Annexin V positive, 7AAD negative MCF-7 cells after 24 h of treatment with 1 μM artemisinin, control (DMSO < 0.01%)μ and plumbagin (5 μM) as positive control. The lower left quadrants of each panels show the viable cells and 7-AAD negative, lower right quadrants represent the early apoptotic cells (PE Annexin V positive and 7-AAD negative). (II) Graph represents the percentage of early apoptotic cells in control and artemisinin treated MCF-7 cells computed from three biologically different set of experiments. Significant differences were observed between control and treated cells, *p < 0.05

The ability of a cancer cell to undergo rapid migration allows it to change position within the tissues. Therapeutic compounds with the ability to inhibit the motility of cancer cells are important for preventing cancer metastasis which may be achieved by a potent drug [67]. Here we have examined the effect of artemisinin on migration of MCF-7 breast cancer cells by wound healing and transwell assay. Monolayer culture of untreated MCF-7 cells, showed 50% reduction in the wound area within 48 h, whereas the reduction in the wound area was significantly less in 1 μM artemisinin treated cells. Artemisinin treated MCF-7 cells migrated at a lower rate and only one quarter of the wound was found to be healed after 96 h, whereas during that interval in untreated MCF-7 cells, about 75% percent of the wound was found to be healed (Fig. 2A I and II). When cancer cells become metastatic, it loses epithelial and gains mesenchymal characteristics which is accompanied by loss of cell-cell adhesiveness, leading to enhanced migratory capacity [68]. Transwell migration assay confirmed the anti-migratory effect of artemisinin on MCF-7 breast cancer cells (Fig. 2B I and II).

One of the major hallmarks of cancer cells is their invasive property. To check the effect of artemisinin on invasive property of breast cancer cells, matrigel migration assay was performed. Significant reduced invasion was evident in MDA-MB-231 aggressive breast cancer cells (Fig. 2C I and II) upon artemisinin treatment.

Apoptosis is as a natural barrier to cancer development and serves as a marker event for chemotherapy [8, 11]. Artemisinin is reported to induce apoptosis in cancer cells. To study the involvement of genes in artemisinin-mediated apoptosis, we first validated the apoptosis inducing effect of artemisinin in MCF-7 breast cancer cells. Annexin V-PE apoptosis detection assay was carried out in control and artemisinin treated MCF-7 breast cancer cells. The flow cytometry data showed 30% increase of PE-Annexin V positive/ 7-AAD negative early apoptotic cells in artemisinin treated with respect to control MCF-7 cells (Fig. 2D I and II).

### Artemisinin-mediated anti-cancerous effects is a result of alteration of relevant genes associated with cancer cell progression

**Fig. 3:**
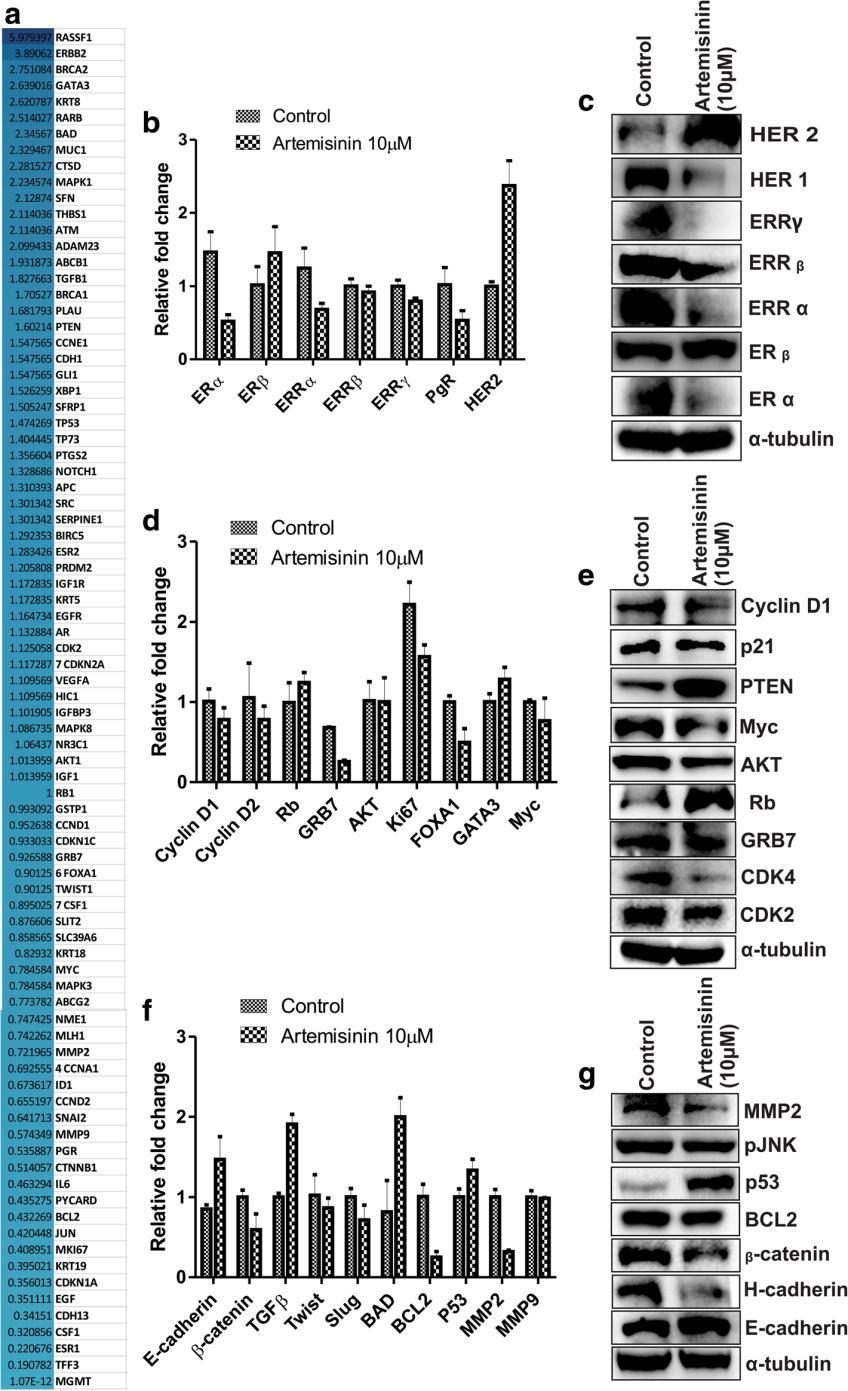
Artemisinin alters the expression of genes associated with growth promoting activities. **a** Heat map showing the fold change expression of genes under study. **b** and **c** qRT PCR and western blot assay respectively showing the expression of genes associated with mammary gland development upon artemisinin treatment. **d** and **e** Respective RNA and protein expression of cell proliferation associated genes in control and artemisinin treated MCF7 cells. **f** and **g** Bar diagram and immunoblot respectively showing the expression level of proteins involved in migration, invasion and apoptosis in artemisinin treated and control cells

In an attempt to reveal the involvement of genes in artemisinin-mediated reduced proliferation, migration, invasion and increased apoptosis, we studied the alteration of genes associated with cancer cell motility upon artemisinin treatment (10 μM, 72 h). In this study PCR array consisting of 84 genes were included. 47 genes out of 84 were found to be upregulated by more than 1.1 fold and reduced expression of 36 genes was observed. 47 upregulated genes included tumor suppressor genes such as well-known BRCA 1, BRCA2, Ras association (RalGDS/AF-6) domain family member 1 (RASSF1), GATA3, RARB, BCL2-associated agonist of cell death (BAD), MUC1 and others as shown in the Fig. 3a with the observed fold change. To validate the alteration in the genes upon artemisinin treatment we checked the expression of genes involved in mammary cell development leading to increased proliferation. Estrogen receptor alpha and beta are well studied for their effect in breast cancer progression. Reduced ERα expression and its associated anti-proliferative effect upon artemisinin is reported [59]. As orphan nuclear receptors ERRα, ERRβ, ERRγ and PgR are significantly involved in increased cancer cell proliferation [69–71], we studied their possible contribution in anti-cancerous effects of artemisinin. Reduced expression of ERRα, ERRβ, ERRγ and PgR was observed in artemisinin treated MCF-7 cells (Fig. 3b and c). At the same time increased expression of tumor suppressor ERβ [72] was found. Although expression of HER family is reported to be down-regulated in artemisinin derivative treated breast cancer cells [36], expression of oncogene HER2 was amplified in artemisinin treated cells at both RNA and protein level but at the same time HER 1 protein expression was reduced significantly. Abrogated expression of oncogenes associated with increased cell proliferation such as cyclin D1, D2 [73] GRB7 [74], AKT [75], Ki67 [76], FOXA1 [77] and Myc [78] was detected. Also, an up-regulated expression of tumor suppressors such as Rb [79], GATA3 [80] and PTEN [81] associated with cancer cell proliferation was evident (Fig. 3d and e). Significantly reduced expression of growth stimulating proteins Cyclin dependent kinases 2 and 4 were found in artemisinin treated cells. At the same time reduced p21 showed its contributing role towards artemisinin mediated reduced cell proliferation validating the previous reports. Epithelial markers such as E-cadherin are key mediators of cell–cell adhesions in epithelial tissues loss of which can promote invasiveness and metastatic behavior in many epithelial tumors [82]. In accordance with this observation, artemisinin treated cells showed enhanced expression of epithelial cell markers E-cadherin, H-cadherin and TGFβ [83], reduced level of mesenchymal proteins Twist and Slug. Reduced level of β-catenin [84] was showed in artemisinin treated cells (Fig. 3f and g). Apoptosis inducing genes BAD and P53 were found to be up regulated whereas anti-apoptotic BCL2 expression was decreased upon artemisinin treatment. Artemisinin mediated reduced invasion is a result of altered MMP2 expression as previously described [85].

### Increased β-catenin cytoplasmic localization contributes toward artemisinin mediated reduced cell migration

**Fig. 4:**
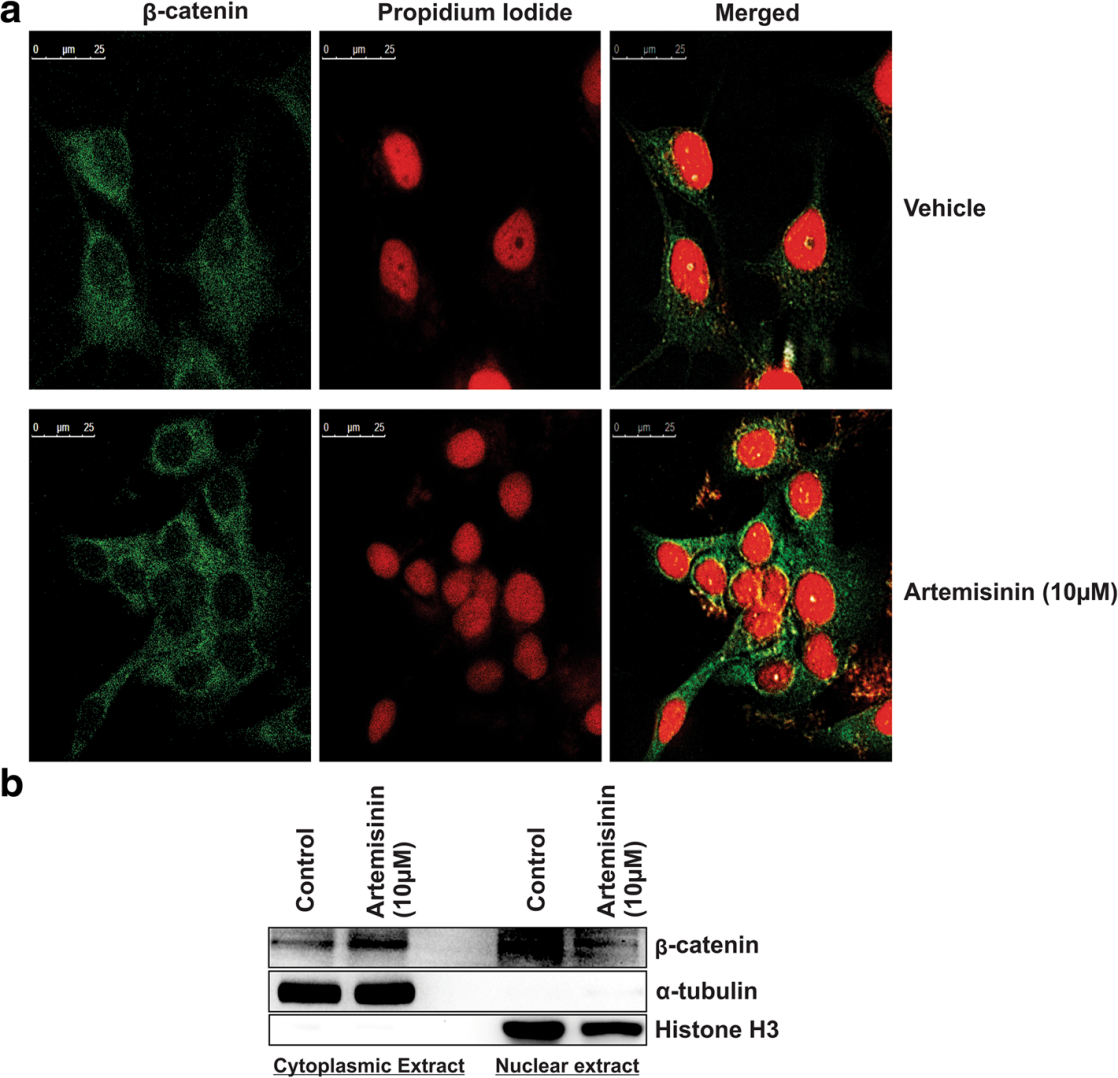
Cytoplasmic localization of Beta-catenin increases upon artemisinin treatment in MCF-7 breast cancer cells. **a** Immunofluorescence assay shows the expression and localization of beta-catenin upon artemisinin treatment. **b** Immunoblot against beta-catenin shows the increased cytoplasmic beta-catenin protein expression upon artemisinin treatment. α-tubulin and histone H3 used as loading control

β-catenin is reported to function as an oncogene through Wnt signaling pathway. Its increased cytoplasmic localization results in reduced gene expression necessary for epithelial to mesenchymal transition. Artemisinin treatment in MCF-7 cells resulted in increased cytoplasmic *β*-catenin protein which indicates its contributing role towards reduced epithelial to mesenchymal transition through Wnt signaling pathway (Fig. 4a and b).

### Increased cytochrome c release and caspase 9 cleavage contributes towards artemisinin mediated increased apoptosis in breast cancer cells

**Fig. 5:**
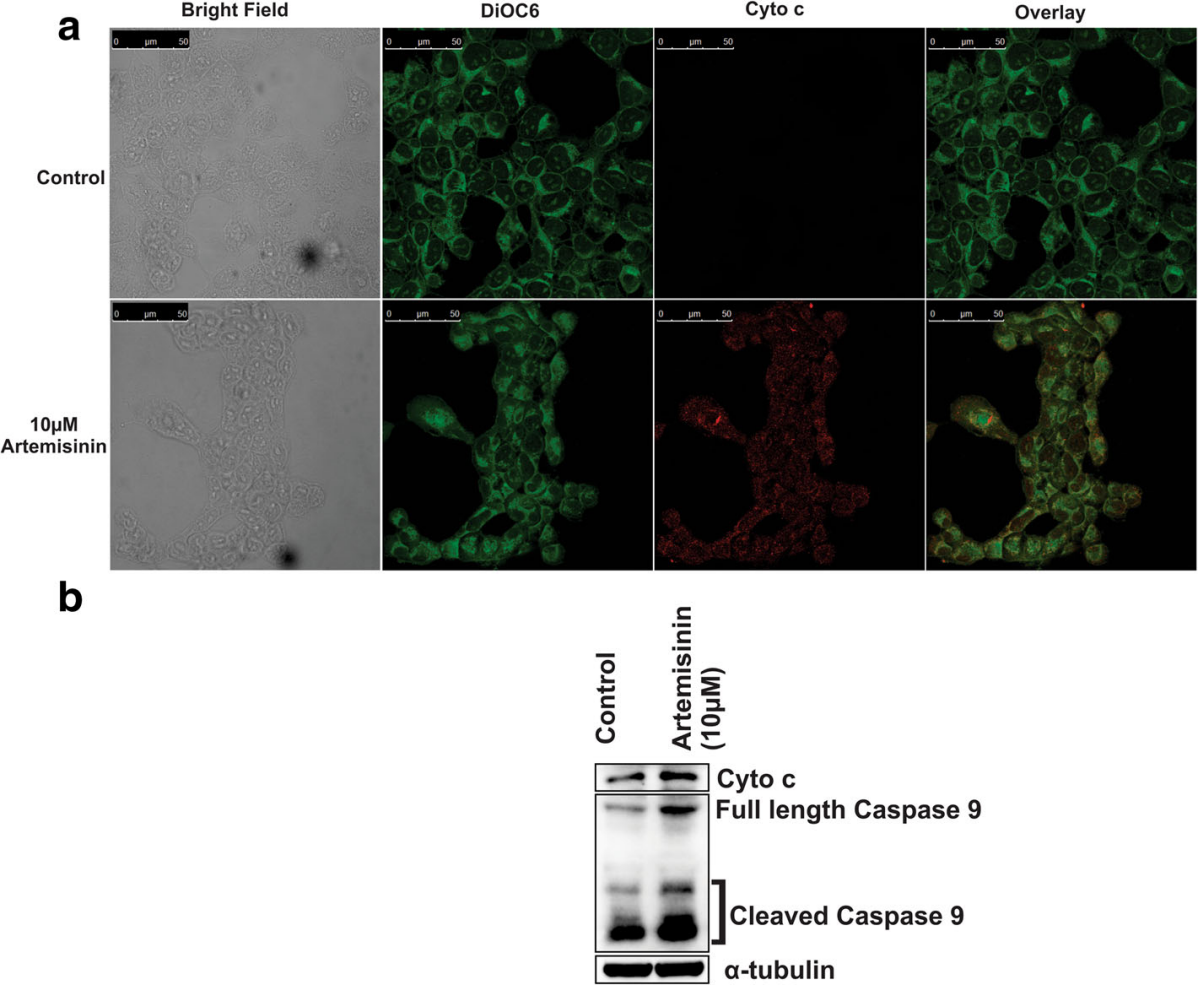
Artemisinin induced apoptosis in MCF-7 cells is also through increased Cytochrome c release and Caspase 9 cleavage. **a** Confocal images of cytochrome c release. Cells stained with mitotracker DiOC6 [91], Cytochrome c (red), merged image shows Cytochrome c release (yellow). **b** Immunoblot against cytochrome c, and caspase 9 showing increased cleaved caspase 9

Cytochrome c is a key component of the electron transport chain that is reported to translocate from the mitochondria to the cytosol in cells undergoing apoptosis. A significant increase in the level of cytochrome c expression was found in artemisinin treated cells as compared to control (Fig. 5a and b). The confocal image showed release of cytochrome c into the cytosol, which seemed to sequential caspase 9 activation. Caspase 9 is an important player in apoptosis. It is an initiator caspase playing important role in programmed cell death [86]. Caspase 9 cleavage acts as an apoptosis marker. Artemisinin treatment resulted in an enhanced cleavage of caspase 9 in MCF-7 breast cancer cells (Fig 5b).

### Artemisinin acts as an inhibitor for histone deacetylases (HDACs)

**Fig. 6:**
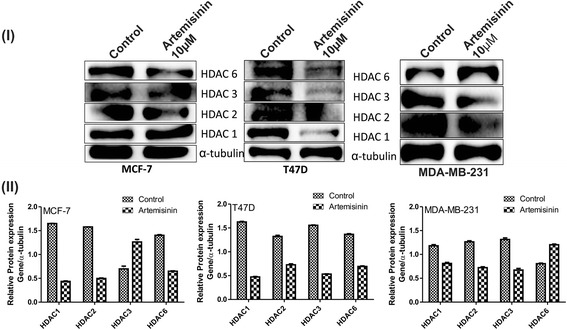
Artemisinin inhibits HDACs. A (I) Western blot assay with protein extracted from MCF-7, T47D and MDA-MB-231 cells treated with artemisinin. Immunoblot was developed using primary antibody HDAC 1, 2, 3 and 6. (II) Densitometry analysis of the protein levels of HDACs as observed in the western blot

Recently, HDAC inhibitors have been investigated as possible target for cancer treatment. While exploring the possible modes of action of artemisinin in cancer cells, we checked the alteration in the expression of epigenetic modifiers HDACs in breast cancer cells upon its treatment. Upon artemisinin treatment, reduced level of HDACs was evidenced. HDAC 1, 2 and 6 were found to be decreased significantly in both the breast cancer cells MCF-7 and T47D (Fig. 6). In MDA-MB-231 cells, HDAC6 level was increased. Expression of HDAC 3 was different in cell types, was found to be increased in MCF-7 at the same time diminished in T47D and MDA-MB-231.

## Discussion

Studies have shown that artemisinin has a potent antimalarial and anticancer activity in many cancer cell lines in vitro [87] and in vivo [88], but its direct role in inhibiting breast cancer cell migration and invasion of breast cancer cells has not been studied in depth. Artemisinin treatment altered the expression of relevant genes involved in mammary gland development, cancer cell proliferation, migration, invasion and apoptosis. Our study shows that a dosage of 1 μM, which is of micromolar range and hence physiologically relevant, causes cancer cell growth inhibition. Migratory behavior of cancer cells have been shown to be related to metastasis, which has always been one of the major challenges in cancer treatment [89], also being one of the key target to improve a patient’s prognosis. Artemisinin induces anti-migratory and reduced invasive effect in breast cancer cells through master regulators such as cadherins and matrix metalloproteinases. Increased β-catenin cytoplasmic localization inhibited EMT in artemisinin treated MCF-7 breast cancer cells. Tumor growth is evident because of uncontrolled proliferation and reduced apoptosis. Thus, reduced proliferation and induction of cancer cell apoptosis is a key strategy in anticancer therapy [90]. Through cyclins and CDKs artemisinin inhibits cell proliferation. Inducing apoptosis contributes to cancer treatment through various mechanisms, inhibiting resistance to immune based cytotoxicity. In current study, role of increased Cytochrome c release and caspase 9 cleavage in artemisinin induced apoptosis was found which validated previous reports suggesting involvement of mitochondrial pathway of apoptosis upon artemisinin treatment in MCF-7 breast cancer cells. Also western blot assay evidenced artemisinin as HDAC inhibitor. HDAC 1, 2 and 6 were significantly reduced upon artemisinin treatment in breast cancer cells.

## Conclusions

Taken together, our data apparently point out to the fact that in response to artimisinin treatment HDACs contributes towards altered expression of tumor suppressor genes and oncogenes resulting into reduced breast cancer cell proliferation, migration, invasion and increased apoptosis. Our data also suggest the role of epigenetics in anti-cancerous activity of artemisinin in cancer. Further exploration is required to establish the contribution of epigenetics in artemisinin-mediated reduced breast tumorigenesis. The obtained findings provide rational insight for the further evaluation of artemisinin as a safe, efficient and selective drug in the treatment and prevention of human breast cancer.

## Abbreviations

7AAD: 7-amino-actinomycin D
DMEM: Dulbecco’s Modified Eagle’s Medium
DMSO: Dimethyl Sulfoxide
EDTA: Ethylenediaminetetraacetic acid
ER: Endoplasmic reticulum
FBS: Fetal Bovine Serum
HRP: Horseradish peroxidase
IC_50_: Median Inhibitory concentration
MTT: 3-(4,5-Dimethylthiazol-2-yl)-2,5-Diphenyltetrazolium Bromide
PBS: Phosphate buffered saline
PE: Phycoerythrin
PVDF: polyvinyl difluoride
RIPA: Radioimmunoprecipitation assay buffer
ROS: Reactive oxygen species
SDS-PAGE: Sodium dodecyl sulfate polyacrylamide gel electrophoresis
TBST: Tris Buffered Saline with Tween® 20
TE: Tris- Ethylenediaminetetraacetic acid
